# Morphological Characteristics, Nutrients, and Bioactive Compounds of *Zizania latifolia*, and Health Benefits of Its Seeds

**DOI:** 10.3390/molecules23071561

**Published:** 2018-06-28

**Authors:** Ning Yan, Yongmei Du, Xinmin Liu, Cheng Chu, John Shi, Hongbo Zhang, Yanhua Liu, Zhongfeng Zhang

**Affiliations:** 1 Tobacco Research Institute of Chinese Academy of Agricultural Sciences, Qingdao 266101, China; duyongmei@caas.cn (Y.D.); liuxinmin@caas.cn (X.L.); chuchengkk@163.com (C.C.); zhanghongbo@caas.cn (H.Z.); liuyanhua@caas.cn (Y.L.); 2 Guelph Food Research Center, Agriculture and Agri-Food Canada, Guelph, ON N1G 5C9, Canada; john.shi@agr.gc.ca

**Keywords:** *Zizania latifolia*, bioactivity, health benefit, Chinese wild rice, swollen culm, aerial parts

## Abstract

*Zizania latifolia* (tribe Oryzeae Dum., subfamily Oryzoideae Care, family Gramineae) is native to East Asian countries. The seeds of *Z. latifolia* (Chinese wild rice) have been consumed as a cereal in China for >3000 years. *Z. latifolia* forms swollen culms when infected with *Ustilago esculenta*, which is the second most-cultivated aquatic vegetable in China. The current review summarizes the nutrients and bioactive compounds of *Z. latifolia*, and health benefits of its seeds. The seeds of *Z. latifolia* contain proteins, minerals, vitamins, and bioactive compounds, the activities of which—for example, antioxidant activity—have been characterized. Various health benefits are associated with their consumption, such as alleviation of insulin resistance and lipotoxicity, and protection against cardiovascular disease. Chinese wild rice may be used to prevent and treat metabolic disease, such as diabetes, obesity, and cardiovascular diseases. Various compounds were isolated from the swollen culm, and aerial parts of *Z. latifolia*. The former suppresses osteoclast formation, inhibits growth of rat glioma cells, and may act as antioxidants and immunomodulators in drugs or foods. The latter exerts anti-fatigue, anti-inflammatory, and anti-allergic effects. Thus, *Z. latifolia* may be used to produce nutraceuticals and functional foods.

## 1. Introduction

*Zizania latifolia* is the sole member of the tribe Oryzeae Dum., subfamily Oryzoideae Care, family Gramineae. It is an important gramineous crop, genetically related to the *Oryza* species [1]. Four *Zizania* species are found worldwide: *Zizania aquatica*, *Zizania palustris*, and *Zizania texana* in North America (U.S.A. and Canada), and *Z. latifolia* in East Asia (China, Japan, and Korea) [2,3]. *Z. aquatica* and *Z. palustris* are annual species, whereas *Z. texana* and *Z. latifolia* are perennial species [3,4]. The genetic diversity of the North American *Zizania* species is higher than that of *Z. latifolia* [5]. The reasons for the low genetic diversity of the latter include restricted gene flow among populations, self-fertilization as a mode of reproduction, and genetic drift within populations [6]. Rivers, mountains, and habitat fragmentation influence the landscape-scale genetic structure of *Z. latifolia* [7]. Molecular evidence indicates that *Zizania* species likely originated in North America, thereafter spreading to East Asia and other parts of the world via the Bering Strait [8].

In contrast to many review articles on the nutritional constituents and health benefits of wild rice (*Zizania* spp.) [3,4], few review articles on the morphological characteristics, nutrients, and bioactive compounds of *Z. latifolia*, and health benefits of its seeds have been published, despite the fact that *Z. latifolia* is valued as a potential source of desirable phytochemicals and nutrients. Therefore, the main objective of the present review was to summarize the current state of knowledge regarding the morphological characteristics of the *Z. latifolia*, the history, present situation, nutrients, bioactive compounds, bioactivities, and health benefits of *Z. latifolia* seeds, and the nutrients and bioactive compounds from the swollen culm and aerial parts of *Z. latifolia*, and to explore the functional food and nutraceutical potential of this plant.

## 2. Methods Used in Searching Research Paper

Our review paper mainly focuses on the morphological characteristics, nutrients, and bioactive compounds of *Z. latifolia*, and health benefits of its seeds. First, we used “*Zizania latifolia*” as the key word to search in the Web of Science, and obtain the related English research papers. Next, we used “*Zizania latifolia*” as the key word to search in the China National Knowledge Infrastructure, and obtained related Chinese research papers. Finally, because of the scarcity of research papers on the bioactive compounds of the seeds of *Z. latifolia*, we have supplemented research papers on the bioactive compounds and health benefits of the seeds of *Z. palustris* and *Z. aquatic*.

## 3. Morphological Characteristics of the *Z. latifolia*

**Figure 1:**
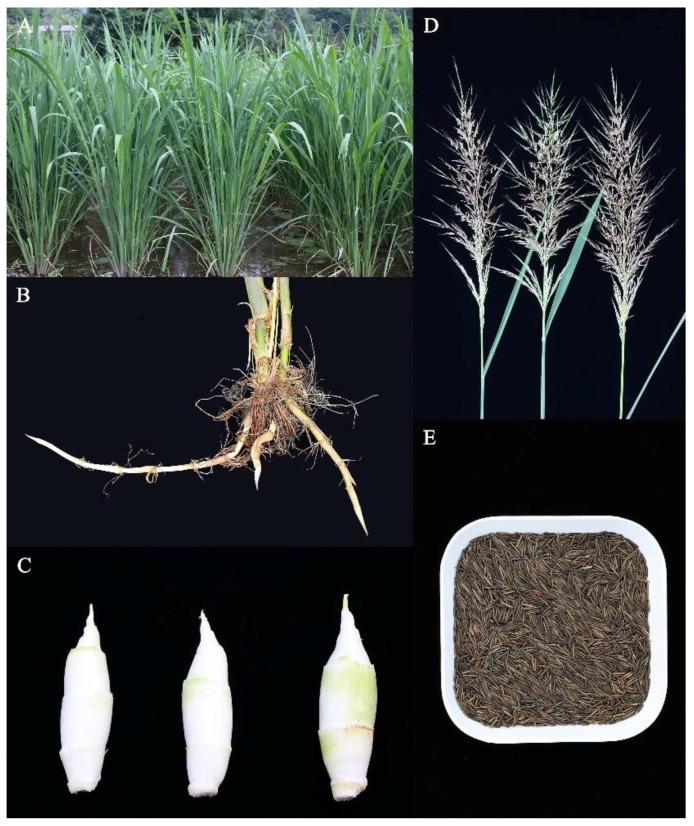
Aerial parts (**A**); rhizome (**B**); swollen culm (**C**); inflorescence (**D**); and seeds (**E**) of *Z. latifolia*.

As a perennial herbaceous plant, *Z. latifolia* grows to 1.5–3.0 m (Figure 1A) and its height increases with water depth [9]. It exhibits high water tolerance and normally grows in clusters on wet marshlands, often with emergent plants such as *Phragmites australis* and *Typha angustifolia* [10,11,12]. *Z. latifolia* produces underground as well as surface stems (rhizomes) that form multiple tillers and enable vegetative propagation (Figure 1B). Infection of *Z. latifolia* by the endophytic smut fungus *Ustilago esculenta* leads to loss of flowers and seeds, with the stem swelling at the base and forming an edible swollen culm (Figure 1C). The swollen culm is the second most-cultivated aquatic vegetable in China, known as *Jiaobai* [13,14,15,16,17]. Asexual propagation via infected rhizomes is the only means of *Jiaobai* production [18]. Long-term artificial selection maximizes the desirable generation of *Jiaobai* and modulates the evolution of the fungus from a plant pathogen to an entophyte [19]. Similar to other endophytic fungi, *U. esculenta* infection does not cause leaf chlorosis or death [14,15,16]. During the period of culm gall formation, *U. esculenta* secretes plant hormones, such as auxin and cytokinin, to stimulate gall formation [20,21]. Environmental factors such as temperature, sunlight, and fertilizer use affect the swollen culm yield [16,22].

*Z. latifolia* not infected by *U. esculenta* forms flowers and seeds under suitable conditions, of which the latter is collected as food [23]. The inflorescence is a 30–50-cm-long panicle that branches out multiple times either upwards or sideways. The plant is monoecious, i.e., female and male flowers are present on the same branch, which are observed among the branches in the middle portion of the inflorescence (Figure 1D). The flowering times vary because of their arrangement on the branch, with the female (located above the male) flowering before the male. The seeds of *Z. latifolia* are sparsely arranged on the ear and mature at different times, after which they fall off easily [23]. They can be deshelled either manually or mechanically to obtain the caryopsis, a cylindrical grain ca. 1.0–1.5 cm long and 1.0–2.0 mm wide, with tapered ends and a light brown color (Figure 1E). Compared to the control (cultivation at 20-cm water depth), cultivation at 50-cm and 100-cm water depths increases the number of inflorescences on *Z. latifolia* [9], which increases the yield of the seeds of *Z. latifolia*.

## 4. History and Present Situation of the *Z. latifolia* Seeds (Chinese Wild Rice)

The *Z. latifolia* seeds have been consumed as a cereal in China for >3000 years, and are one of the earliest cultivated crops. The earliest record of the species comes from the annals of the Zhou Dynasty. ‘*Zhou Li*’, a classical book of ancient China, lists Chinese wild rice as one of the ‘six grains’ (the other five being rice, broomcorn millet, panicled millet, wheat, and beans) offered to the emperor for his enjoyment [24]. However, the habitat of *Z. latifolia* has changed drastically with the rapid increase of the Southern Chinese population and the drying of lakes for rice cultivation, both of which have reduced the area occupied by this species. It has gradually been outnumbered by regular rice and is consumed less frequently, mostly for relieving hunger. Currently, it is rarely cultivated as a crop and mainly survives untended in the wild. It should be noted that *Z. latifolia* is prevalent throughout China, except in Xinjiang and Tibet, and grows in lakes, ponds, rivers, marshes, and the wetlands [6,7]. It is particularly abundant in the middle and lower Yangtze River basin and Huai River basin [5,25,26]. A *Z. latifolia* survey of 10 lakes of the Eastern China plain over 5 consecutive years revealed growth over a 488-km^2^ area, with the generation of ca. 1.8 × 10^7^ kg seeds and 5.5 × 10^8^ kg aerial parts [26]. Between 1981 and 2009, the area covered by *Z. latifolia* in the Wuchang Lake increased linearly, with 53% of the lake surface occupied by 2009; this overgrowth was mainly associated with changes in the water level [27].

## 5. Nutrients and Bioactive Compounds of Chinese Wild Rice

### 5.1. Nutrients of Chinese Wild Rice

The *Z. latifolia* seed (Chinese wild rice) is a type of whole grain with high nutritional value; it contains proteins, minerals, and vitamins, as well as bioactive compounds [3,4,28]. According to laboratory and population-based studies, Chinese wild rice is a safe and unique tasting food, the consumption of which shows a protective effect [24]. The nutrients of Chinese wild rice are shown in Table 1. The vitamin (vitamin B1, vitamin B2, and vitamin E) content in Chinese wild rice from five lakes of Jiangsu Province is appreciably higher than in polished *Indica* rice from Jiangsu Province [29]. In addition, it contains higher amounts of essential amino acids than white rice, barley, and maize [30,31]. The amino acid score of Chinese wild rice is 66.6, which is considerably higher than that of polished *Indica* rice (51.1) [28]. The first and second limiting amino acids of Chinese wild rice are originally considered to be threonine and lysine, respectively [31]. However, a subsequent study by Jiang et al. [28] shows that lysine and isoleucine are the first and second limiting amino acids of Chinese wild rice, respectively. The protein efficiency ratio of Chinese wild rice is 2.75, which is higher than that of enriched white flour (0.6), rice (2.18), and soybean (2.32) [29]. Therefore, proteins derived from Chinese wild rice are considered to be high quality.

The lipid content of North American wild rice ranges from 0.7 to 1.1%, compared to 2.7% in regular brown rice [35]. The main lipids in North American wild rice are linoleic (35–37%), linolenic (20–31%), palmitic (14.1–18.4%), and oleic (12.8–16.2%) acids [35]. The amount of moisture, protein, fat, ash, and dietary fiber in Chinese wild rice is similar to that in North American wild rice; however, iron, vitamin B1, and vitamin E content of Chinese wild rice is higher, whereas methionine, zinc, and vitamin B2 content is lower [30,31]. The amount of vitamin B1 in Chinese wild rice is 0.52–0.63 mg/100 g, whereas that in North American wild rice is 0.36–0.50 mg/100 g; the total vitamin E content of North American wild rice is reportedly 0.2 mg/100 g lipids, whereas it is 0.48 mg/100 g lipids in Chinese wild rice [31]. Although the total carbohydrate content of Chinese wild rice is lower than that in polished *Indica* rice, the levels of other components and elements are higher, including protein (2.16-fold), fat (1.54-fold), total dietary fiber (7.62-fold), and total mineral levels (1.91-fold), as well as calcium (3.32-fold), chromium (4.00-fold), copper (1.83-fold), iron (2.39-fold), magnesium (3.14-fold), manganese (1.31-fold), phosphorus (2.93-fold), potassium (2.83-fold), sodium (1.57-fold), and zinc (1.39-fold) [28]. Therefore, Chinese wild rice is a rich source of minerals (Table 1).

### 5.2. Bioactive Compounds in Chinese Wild Rice

In addition to nutrients, Chinese wild rice also contains bioactive compounds (Table 1). The flavonoid, saponin, and phytosterol content in Chinese wild rice are 52.25-, 12.12-, and 4.29-times higher, respectively, than in white rice [28]. Importantly, anthocyanins and chlorophyll, which were not detected in white rice, are present in high quantities in Chinese wild rice. Although the bioactive compounds of Chinese wild rice have not been extensively studied, relatively more data are available regarding the flavonoid, phenolic acid, cell wall hydroxycinnamate, γ-oryzanol, and phytosterol of North American wild rice [35,36,37,38,39]. Among these, the total phenolic content of North American wild rice varies from 2472 to 4072 mg of ferulic acid equivalents (FAE)/kg, which is higher than that in white rice (279 mg of FAE/kg) [38]. Flavonoid glycosides (diglucosyl apigenin, glucosyl-arabinosyl apigenin, and diarabinosyl apigenin) and flavan-3-ols (catechin, epicatechin, and oligomeric procyanidin) are the main antioxidants identified in North American wild rice [38]. Ferulic acid (FA) is the most abundant phenolic acid (up to 355 mg/kg), followed by sinapic acid, in North American wild rice; the other monomeric phenolic acid compounds present in North American wild rice are *p*-coumaric acid, vanillic acid, syringic acid, *p*-hydroxybenzoic acid, *p*-OH-benzaldehyde, and vanillin [39]. Phenolic acid dehydrodimers are cell wall-bound, and are only detectable in the methanol-insoluble fractions; they are represented by diferulic and disinapic acids [39]. FA is the most abundant phenolic acid (394.2 mg/kg) among the insoluble dietary fibers in North American wild rice, although significant amounts of sinapic acid (51.8 mg/kg) and *p*-coumaric acid (14.2 mg/kg) were also detected [37]. Five feruloylated oligosaccharides were isolated and identified as arabinoxylan ferulate fragments, and the new feruloylated tetrasaccharide {[5-*O*-(*trans*-feruloyl)][*O*-*β*-d-Xyl*p*-(1→2)]-*O*-*α*-l-Ara*f*-(1 → 3)}-*O*-*β*-d-Xyl*p*-(1 → 4)-d-Xyl*p* was isolated from North American wild rice [37]. North American wild rice contains more γ-oryzanol, a natural mixture of FA esters of triterpene alcohols and sterols, and an important bioactive component in rice bran oil, than regular brown rice (1352 vs. 688 mg/kg); cycloartenol ferulate is the most abundant sterol ferulate in North American wild rice, whereas 24-methylenecycloartenol ferulate is the most abundant in regular brown rice [36]. There are more saturated ferulates in regular brown rice compared to North American wild rice; four additional γ-oryzanol compounds are identified as caffeates and cinnamates of cycloartenol and campesterol in North American wild rice [36]. To our knowledge, North American wild rice is a source of bioactive phytosterols; the main sterols of North American wild rice found in an unsaponified fraction were: campesterol (14–52%), *β*-sitosterol (19–33%), Δ^5^-avenasterol (5–12%), and cycloartenol (5–12%) [35]. Moreover, the level of omega-3 fatty acids in North American wild rice is up to 18 times higher than in brown rice [35]. Anthocyanins are thought to be the major functional components in pigmented rice [40], which may be the major functional components in North American wild rice [38]. The bioactivities and health benefits of wild rice may be attributed to the synergistic effects of its functional components. Notably, the inhibitory effects of dietary polyphenols against α-glucosidases, α-amylases, and aldose reductases have attracted interest among researchers [41,42,43].

## 6. Bioactivity and Health Benefits of Chinese Wild Rice

### 6.1. Antioxidant Activity

Whole grains are rich in bioactive substances, such as antioxidants, which protect the body against oxidative stress [44]. As a whole-grain food, Chinese wild rice contains high levels of antioxidants [28], which can effectively remove free radicals from the body, and enhance the function of antioxidant systems. When incorporated into different meat products, wild rice retards lipid oxidation, with phytic acid being one of the potent antioxidants of wild rice [45]. The antioxidant activity of raw *Z. palustris* seeds is 30 times higher than that of control white rice, and the total phenolic content is highly correlated with the total antioxidant activity of *Z. palustris* seeds [38]. The diphenylpicrylhydrazyl (DPPH) free radical-scavenging activities of the soluble and insoluble phenolic acids suggest that the antioxidant activity of *Z. palustris* seeds is partially associated with its phenolic acid profile [39]. The main contributors to the antioxidant activity of free flavonoids in *Z. aquatica* seeds are epigallocatechin, epicatechin, and rutin, and the main contributors to the antioxidant activity of free phenolic acids in *Z. aquatica* seeds are ferulic, vanillic, ellagic, sinapic, and syringic acids [46]. Chinese wild rice also contains the trace elements selenium and copper, as well as vitamin E, which contribute to its enzymatic and non-enzymatic antioxidant activities [29]. Consumption of Chinese wild rice prevents the accumulation of oxidative stress in rats fed a high saturated fat and cholesterol diet; this is because of increased antioxidant and superoxide dismutase activities, and reduced malondialdehyde concentration in the serum and liver [47,48] (Table 2). Owing to its superior antioxidant capacity, Chinese wild rice can inhibit the deleterious effects of lipid peroxidation to ameliorate chronic metabolic diseases.

### 6.2. Alleviation of Insulin Resistance and Lipotoxicity

Chinese wild rice is abundant in dietary fiber, resistant starch, and phytosterols, as well as polyphenols, such as flavonoids, saponins, and anthocyanins [28] (Table 1). Replacing white rice and processed wheat starch with Chinese wild rice as the main source of dietary carbohydrate in rats fed high saturated fat and cholesterol diet ameliorated abnormal glucose metabolism by suppressing diet-induced insulin resistance [49,51]. Animal studies revealed that Chinese wild rice prevented increase in body weight and fat accumulation in insulin-resistant mice, and lowered the serum levels of glucose, insulin, and free fatty acids [49]. Chinese wild rice inhibits the expression of protein tyrosine phosphatase 1B in the liver to promote the phosphorylation of insulin receptor substrate-2, thereby enhancing insulin signal transduction and reducing insulin resistance (Table 2). Dietary carbohydrate replaced with Chinese wild rice reduced triglyceride and free fatty acid levels in the liver, leading to increase in serum adiponectin levels, reduction in serum lipocalin-2 and visfatin concentrations, up-regulation of the expressions of adiponectin receptor 2, peroxisome proliferator-activated receptor alpha, and gamma, and down-regulation of leptin and lipocalin-2 expression [51] (Table 2). Because of its low glycaemic index, Chinese wild rice alleviated insulin resistance in rats induced by high saturated fat and cholesterol diet when it was used to replace 50% of the enriched rice and flour content in the diet [50]. The total dietary fiber content of Chinese wild rice is 7.62 times that of polished *Indica* rice; its resistant starch content (11.73 g/100 g) is significantly higher than that of rice flour (7.71 g/100 g) and wheat flour (7.79 g/100 g) [28]. The ratios of omega-6 to omega-3 fatty acids in *Z. palustris* seeds range from 1.1 to 1.8, whereas those in regular brown rice range between 20.2 and 22.4 [35]. Reports indicate the beneficial effects of the lower ratios of omega-6 to omega-3 fatty acids in North American wild rice on human blood lipid level. In rats fed high saturated fat and cholesterol diet, Chinese wild rice effectively reduced fat deposition, and lowered serum total cholesterol, triglyceride, tumor necrosis factor (TNF)-α, C-reactive protein, and free fatty acid levels; in contrast, it increased high-density lipoprotein cholesterol levels by enhancing lipid metabolism. Lipotoxicity is a metabolic syndrome that results from the accumulation of lipid intermediates in non-adipose tissue, leading to cellular dysfunction and death, and is involved in heart failure, obesity, and diabetes [56]. Notably, Chinese wild rice reduced hepatic lipotoxicity, lowered blood lipid levels in rats with dysregulated lipid metabolism, and reduced low-level inflammation caused by hyperlipidaemia [52,53]. It also prevented the up-regulation of sterol-regulatory element binding protein-1c, fatty acid synthase, and acetyl-CoA carboxylase, and down-regulated lipoprotein lipase and adipose triglyceride lipase induced by high saturated fat and cholesterol diet [54] (Table 2). Therefore, Chinese wild rice belongs to foods of low glycaemic index, which can alleviate insulin resistance and lipotoxicity.

### 6.3. Cardiovascular Disease Prevention

Soluble dietary fiber can reduce blood glucose levels and bind to bile acid for excretion in feces, thereby lowering cholesterol levels and reducing the risk of heart disease, atherosclerosis, and other conditions associated with high cholesterol levels [52]. Owing to its high resistant starch content, Chinese wild rice lowers cholesterol and blood lipid levels, which is associated with a reduced incidence of cardiovascular diseases, including coronary heart disease and atherosclerosis [57]. Consumption of *Z. palustris* seeds appreciably reduced the size and severity of atherosclerotic lesions in the aortic roots of male and female low-density lipoprotein receptor-knockout (LDLr-KO) mice, by 71% and 61%, respectively, compared to the gender-matched controls [58]. The phytosterol content of Chinese wild rice (71.28 mg/100 g) was 4.29 times higher than that of polished *Indica* rice [28]. Consumption of 60% (*w*/*w*) *Z. palustris* seeds in combination with 2% (*w*/*w*) phytosterols appreciably reduced the size and severity of atherosclerotic lesions in the aortic roots of LDLr-KO mice compared to the control group [59]. This effect was associated with an appreciable reduction of total plasma, low-density lipoprotein, and very low-density lipoprotein cholesterol levels, as well as increase in fecal cholesterol excretion. Chinese wild rice lowered blood cholesterol concentration in rats with high lipid levels, and inhibited the formation of atherosclerotic plaques and the occurrence of fatty liver, while simultaneously increasing antioxidant activity in serum and suppressing inflammation associated with atherosclerosis [55] (Table 2). When compared to white rice, *Z. palustris* seeds inhibit monocyte adhesion to the aorta, atherosclerosis, and the concentration of inflammatory and fibrinolytic regulators in the cardiovascular tissue of LDLr-KO mice [60]. The anti-atherosclerotic effect of wild rice in LDLr-KO mice may be associated with its inhibition of monocyte adhesion and inflammatory modulators [60].

## 7. Nutrients and Bioactive Compounds of the Swollen Culm of *Z. latifolia*

The swollen culm, induced by the smut fungus *U. esculenta*, is consumed as an important aquatic vegetable in China, Japan, and other Asian countries [13,14,15,16,61,62]. Nutrients in the swollen culm of *Z. latifolia* are shown in Table 1. Qian et al. [32] determined the nutrient content of the swollen culm of *Z. latifolia* to be as follows: moisture, 92.001 g/100 g fresh weight (FW); protein, 2.018 g/100 g dry weight (DW); fat, 2.258 g/100 g FW; starch, 1.429 g/100 g DW; total soluble sugars, 35.719 g/100 g DW; reducing sugars, 30.854 g/100 g DW; ascorbic acid, 0.60 g/100 g DW; polyphenols, 1.050 g/100 g FW; ash, 0.531 g/100 g DW; total dietary fiber, 4.220 g/100 g FW; soluble dietary fiber, 0.700 g/100 g FW; insoluble dietary fiber, 3.510 g/100 g FW; lignin, 0.567 g/100 g FW. Another study reported the following nutrient composition of the swollen culm of *Z. latifolia*: carbohydrate, 13.82%; protein, 8.13%; fat, 1.0%; lignin, 7.0%; sodium, 0.02%; iron, 0.85 ppm; magnesium, 3.34 ppm; copper, 0.12 ppm; zinc, 4.71 ppm; and arsenic, 0.09 ppm [63]. These reported differences between nutrients in the swollen culm of *Z. latifolia* may be associated with crop variety, growth environment, cultivation techniques, and harvest time.

**Figure 2:**
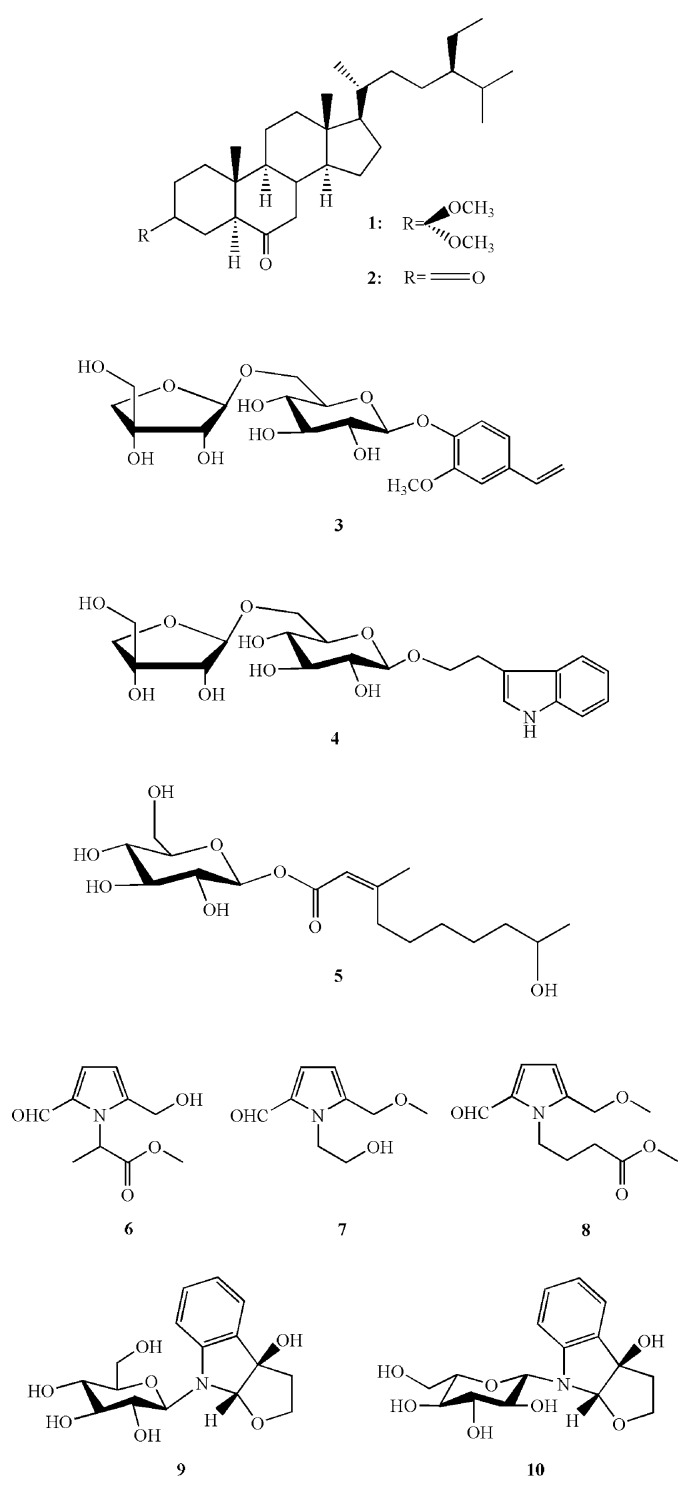
Chemical structures of compounds (**1**–**10**) isolated from the swollen culm of *Z. latifolia*.

Various compounds (**1**–**10**, Figure 2) have been isolated from the swollen culm of *Z. latifolia* [64,65,66], including osteoclast-inhibiting compounds **1** (white crystal) and **2** (colorless amorphous crystal). The combination of **1** (25 μg/mL, 53 μM) and **2** (25 μg/mL, 58 μM) reduced the respective number of TRAP-(+) multinucleated cells to 49% and 19% without cytotoxicity [65]. Makomotine A (**3**, white amorphous powder), makomotine B (**4**, yellow amorphous powder), makomotine C (**5**, white amorphous powder), makomotine D (**6**, colorless oil), and two other compounds (**7**, **8**) were also isolated from the swollen culm; 25 μg/mL of **3** inhibited osteoclast formation without cytotoxic effects in vitro [64]. The l-glucoside isomer (**10**) of makomotindoline (**9**, colourless amorphous solid) reduced the number of rat glioma cells after 24 h when applied at a concentration of 10 μM (92.4 ± 9.1%) and 100 μM (83.9 ± 8.0%) [66]. Water- and alkali-extractable polysaccharides (ZLPs-W and ZLPs-A, respectively) were recently extracted from the swollen culms of *Z. latifolia* [67]. Both compounds exhibit radical-scavenging activity; the half-maximal effective concentrations of the DPPH, superoxide radical-, and hydroxyl radical-scavenging activities of ZLPs-A were 1.87, 1.13, and 0.38 mg/mL, respectively, whereas the corresponding values for ZLPs-W were 2.95, 3.99, and 0.5 mg/mL, respectively. ZLPs-W show no cytotoxicity, and their immunomodulatory activity is higher than that of ZLPs-A, as indicated by higher phagocyte stimulation and nitric oxide (NO) production in RAW 264.7 macrophages [67]. ZLPs-W were further separated into three purified polysaccharides (ZLPs-W1, ZLPs-W2, and ZLPs-W3), which effectively enhanced the proliferation, phagocytosis, and NO production of RAW 264.7 macrophages, suggesting that they possess a potent immunostimulatory activity and might be developed as immunomodulators in drugs or foods [68].

## 8. Nutrients and Bioactive Compounds of the Aerial Parts of *Z. latifolia*

Although the aerial part of *Z. latifolia* is not eaten directly as food, several studies have reported the main nutrients contained therein. Qian et al. [32] reported the following nutrients in the leaf sheath: moisture, 870.00 g/kg FW; protein, 11.71 g/kg DW; fat, 18.10 g/kg FW; starch, 25.86 g/kg DW; total soluble sugars, 220.53 g/kg DW; reducing sugars, 182.73 g/kg DW; ascorbic acid, 0.01 g/kg DW; polyphenols, 6.30 g/kg FW; ash, 10.10 g/kg DW; total dietary fiber, 106.20 g/kg FW; soluble dietary fiber, 28.90 g/kg FW; insoluble dietary fiber, 77.40 g/kg FW; lignin, 8.19 g/kg FW. Another study reported the biomass chemical composition of the aerial parts of *Z. latifolia* to be as follows: cellulose, 28.5%; hemicellulose, 12.9%; lignin, 31.0%; ash, 3.0%; extractives, 18.9% [2]. Considering their high protein, cellulose, hemicellulose, and lignin content, the aerial parts of *Z. latifolia* could be used as animal feed, culture material for edible mushrooms, feed stock for bioethanol production, and activated carbon [2,32,69].

**Figure 3:**
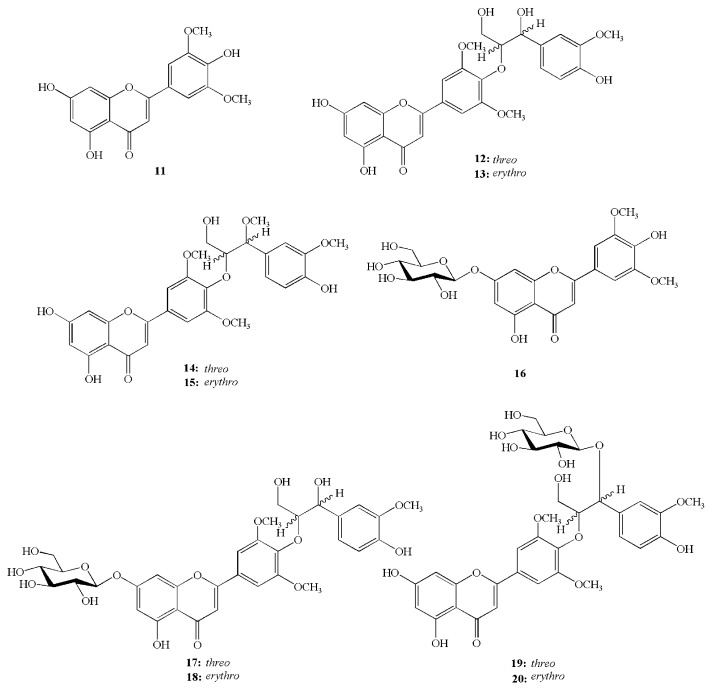
Chemical structures of compounds (**11**–**20**) isolated from the aerial parts of *Z. latifolia*.

The aerial parts of *Z. latifolia* have been shown to exert anti-fatigue, anti-inflammatory, and anti-allergic effects [70,71,72,73,74]. A methanol extract of the aerial parts of *Z. latifolia* inhibits compound 48/80-induced degranulation, antigen-induced β-hexosaminidase release, and PMA plus A23187-induced TNF-α production in RBL-2H3 mast cells [71]. In contrast, the chloroform fraction of the plant tissue suppresses allergic inflammatory response by blocking the release of β-hexosaminidase and TNF-α from RBL-2H3 cells stimulated with dinitrophenyl and bovine serum albumin, and inhibiting the expression of cyclooxygenase-2 and activation of the mitogen-activated protein kinase [74]. Tricin (**11**, pale yellow powder), salcolin A (**12**, yellow amorphous powder), salcolin B (**13**, yellow amorphous powder), salcolin C (**14**, yellow amorphous powder), and salcolin D (**15**, yellow amorphous powder) were recently isolated from the aerial parts of *Z. latifolia* (Figure 3). Compounds **12**–**15** are tricin derivatives with more potent anti-inflammatory and anti-allergic activities than tricin (**11**) [72]. In particular, compound **15** strongly inhibits lipopolysaccharide-induced NO production in RAW 264.7 cells, as well as β-hexosaminidase release in IgE-sensitized RBL-2H3 cells. Other compounds isolated from the aerial parts of *Z. latifolia* include tricin-7-*O*-β-d-glucopyranose (**16**, pale yellow powder), tricin-4′-*O*-(threo-β-guaiacylglyceryl) ether 7-*O*-β-d-glucopyranose (**17**, yellow amorphous powder), tricin-4′-*O*-(erythro-β-guaiacylglyceryl) ether 7-*O*-β-d-glucopyranose (**18**, yellow amorphous powder), tricin-4′-*O*-(threo-β-guaiacylglyceryl) ether 7″-*O*-β-d-glucopyranose (**19**, yellow amorphous powder), and tricin-4′-*O*-(erythro-β-guaiacylglyceryl) ether 7″-*O*-β-d-glucopyranose (**20**, yellow amorphous powder) [73] (Figure 3). To develop and use these bioactive compounds, they must be isolated from the aerial parts of *Z. latifolia*.

## 9. Conclusions

Chinese wild rice contains proteins, minerals, vitamins, and bioactive substances. It exhibits various bioactivities, including antioxidant activity. A number of health benefits are associated with its consumption, e.g., alleviation of insulin resistance and lipotoxicity, and protection against cardiovascular disease. Therefore, Chinese wild rice may be used to prevent and treat metabolic disease, such as obesity, diabetes, and cardiovascular diseases. However, no human dietary intervention studies have been performed, although studies where human subjects will be administered realistic doses of Chinese wild rice as a part of their daily diet are required. Although considerable progress has been made toward understanding the bioactivities and health benefits of Chinese wild rice, relatively fewer studies focus on the bioactivities and structure-activity relationship of the isolated compounds. Consequently, future studies will focus on the isolation of compounds with high bioactivities and stable chemical structures, and elucidate their structure-activity relationship. Compounds from the swollen culm of *Z. latifolia* have been shown to suppress osteoclast formation and inhibit the growth of glioma cells. Their role as potential antioxidants and immunomodulators for use in drugs or food products have also been investigated. Furthermore, compounds from the aerial parts of *Z. latifolia* can be used to treat fatigue, inflammation, and allergic reaction.

## Figures and Tables

**Table 1:** Nutrients and bioactive compounds of the seed and swollen culm of *Z. latifolia*.

Unit per 100 g	Seed [28,31]	Swollen Culm [32,33,34]
Moisture (g)	9.51 ± 0.13	92.001 ± 0.90
Protein (g)	13.30 ± 1.38	2.818 ± 0.019
Fat (g)	1.08 ± 0.12	2.258 ± 0.071
Total carbohydrates (g)	73.18 ± 1.68	2.43 ± 0.19
Total dietary fibre (g)	7.24 ± 1.29	4.22 ± 0.006
Total minerals (g)	1.30 ± 0.04	0.531 ± 0.001
Vitamin B1 (mg)	0.59 ± 0.04	0.02
Vitamin B2 (mg)	0.11 ± 0.03	–
Vitamin E (mg)	0.29 ± 0.11	0.99
Alanine (g)	0.69 ± 0.07	0.06 ± 0.008
Arginine (g)	1.13 ± 0.13	0.04 ± 0.005
Aspartic acid (g)	1.19 ± 0.15	0.15 ± 0.028
Cysteine (g)	0.37 ± 0.02	–
Glutamic acid (g)	2.40 ± 0.24	0.11 ± 0.010
Glycine (g)	0.59 ± 0.06	0.04 ± 0.005
Histidine (g)	0.38 ± 0.03	0.05 ± 0.005
Isoleucine (g)	0.50 ± 0.05	0.04 ± 0.003
Leucine (g)	0.95 ± 0.10	0.07 ± 0.007
Lysine (g)	0.66 ± 0.07	0.07 ± 0.007
Methionine (g)	0.28 ± 0.01	0.01 ± 0.000
Phenylalanine (g)	0.65 ± 0.08	0.04 ± 0.005
Proline (g)	0.37 ± 0.05	0.04 ± 0.005
Serine (g)	0.66 ± 0.07	0.06 ± 0.007
Threonine (g)	0.44 ± 0.04	0.05 ± 0.007
Tryptophan (g)	0.21 ± 0.03	–
Tyrosine (g)	0.44 ± 0.05	0.03 ± 0.003
Valine (g)	0.70 ± 0.08	0.05 ± 0.005
Calcium (mg)	23.74 ± 0.47	4.00
Chromium (mg)	0.12 ± 0.03	–
Copper (mg)	0.22 ± 0.13	–
Iron (mg)	2.80 ± 0.27	0.40
Magnesium (mg)	114.74 ± 7.21	8.00
Manganese (mg)	1.34 ± 0.11	0.49
Phosphorus (mg)	291.20 ± 37.64	36.00
Potassium (mg)	218.47 ± 11.06	209.00
Sodium (mg)	4.48 ± 0.87	5.80
Zinc (mg)	2.40 ± 0.20	0.33
Flavonoids (mg)	352.00 ± 3.12	383.70
Saponins (mg)	354.11 ± 22.70	–
Anthocyanins (mg)	258.00 ± 17.31	–
Chlorophyll (mg)	108.40 ± 2.41	–
Phytosterols (mg)	71.28 ± 8.12	–

**Table 2:** Bioactivity and health benefits of *Z. latifolia* seeds (Chinese wild rice).

Bioactivity or Health Benefit	Putative Functional Compounds	Potential Mechanism	Reference
Antioxidant activity	Vitamins, minerals, phytosterols, phenolic acid, phytic acid, flavonoids, saponins, anthocyanins, and chlorophyll	Suppressing oxidative stress by increasing antioxidant capacity and superoxide dismutase activity, and reducing malondialdehyde concentration	[47,48]
Alleviation of insulin resistance and lipotoxicity	Dietary fibre, resistant starch, vitamins, minerals, polyunsaturated fatty acids, phytosterols, phenolic acids, flavonoids, saponins, anthocyanins, and chlorophyll	(1) Improving glucose metabolism and insulin sensitivity by inhibiting the expression of protein tyrosine phosphatase 1B and enhancing the expression of insulin receptor substrate-2 in the liver (2) Decreasing triglyceride and free fatty acid levels in a liver homogenate; increasing adiponectin, and decreasing lipocalin-2 and visfatin concentrations in the serum (3) Increasing the expression of adiponectin receptor 2, and peroxisome proliferator-activated receptors alpha and gamma; inhibiting the expression of leptin and lipocalin-2 (4) Improving lipid metabolism and liver lipotoxicity, and suppressing low-grade inflammation induced by a high saturated fat and cholesterol diet in hyperlipidaemic rats (5) Suppressing increase in lipid droplet accumulation, and free fatty acid and leptin concentrations; reducing lipoprotein lipase and adipose triglyceride lipase levels (6) Inhibiting expression of sterol-regulatory element binding protein-1c, and fatty acid synthase and acetyl-CoA carboxylase induced by high saturated fat and cholesterol diet	[49,50,51,52,53,54]
Cardiovascular disease prevention	Dietary fibre, resistant starch, vitamins, phytosterols, phenolic acids, flavonoids, saponins, anthocyanins, and chlorophyll	(1) Reducing blood lipid levels in rats fed high-fat diet, and inhibiting the formation of atherosclerotic plaques and the occurrence of fatty liver (2) Enhancing antioxidative capacity and preventing atherosclerosis	[55]
